# Predicting the locations of force-generating dyneins in beating cilia and flagella

**DOI:** 10.3389/fcell.2022.995847

**Published:** 2022-10-11

**Authors:** Jonathon Howard, Alexander Chasteen, Xiaoyi Ouyang, Veikko F. Geyer, Pablo Sartori

**Affiliations:** ^1^ Department of Molecular Biophysics & Biochemistry, Yale University, New Haven, United States; ^2^ Instituto Gulbenkian de Ciência, Oeiras, Portugal; ^3^ Department of Physics, Yale University, New Haven, United States; ^4^ Yale Quantitative Biology Institute, New Haven, United States; ^5^ Center for Molecular and Cellular Bioengineering (CMCB), Technische Universität Dresden, Dresden, Germany

**Keywords:** dynein, axoneme, cilium, flagellum, force

## Abstract

Cilia and flagella are slender cylindrical organelles whose bending waves propel cells through fluids and drive fluids across epithelia. The bending waves are generated by dynein motor proteins, ATPases whose force-generating activity changes over time and with position along the axoneme, the motile structure within the cilium. A key question is: where, in an actively beating axoneme, are the force-generating dyneins located? Answering this question is crucial for determining which of the conformational states adopted by the dynein motors generate the forces that bend the axoneme. The question is difficult to answer because the flagellum contains a large number of dyneins in a complex three-dimensional architecture. To circumvent this complexity, we used a molecular-mechanics approach to show how the bending moments produced by single pairs of dynein motors work against elastic and hydrodynamic forces. By integrating the individual motor activities over the length of the axoneme, we predict the locations of the force-generating dyneins in a beating axoneme. The predicted location depends on the beat frequency, the wavelength, and the elastic and hydrodynamic properties of the axoneme. To test these predictions using cryogenic electron microscopy, cilia with shorter wavelengths, such as found in *Chlamydomonas*, are more suitable than sperm flagella with longer wavelengths because, in the former, the lag between force and curvature is less dependent on the specific mechanical properties and experimental preparation.

## Introduction

**FIGURE 1:**
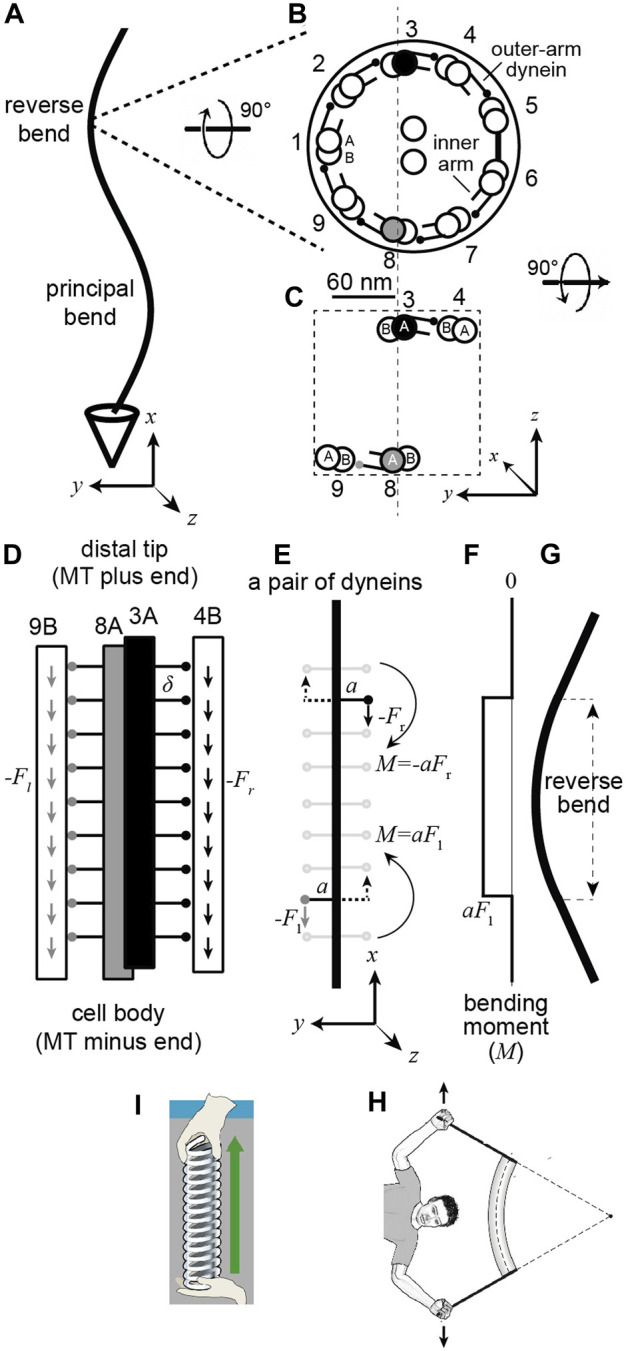
A pair of dynein motor proteins can generate a bend. (**A)** A flagellum with the right-handed coordinate system (axes drawn below). (**B)** The cross-section when viewed from the base with the standard numbering (Afzelius 1988) and the dynein arms pointing clockwise. The center-to-center spacing of the doublets is approximately 60 nm. The small circles at the ends of the lines emerging from the complete A-tubule represent the microtubule-binding domains (MTB) of the outer-arm dyneins (just one MTB is shown, though sperm have two and *Chlamydomonas* has three). **(C)** A pair of dyneins on opposite sides of the axonemal section. **(D)** view along the length of the axoneme showing the outer-arm dyneins spaced every 24 nm ( δ ) and generating downward forces (towards the minus ends of the microtubules). (**E)** The upper right dynein generates a negative moment and the lower left one a positive moment. The dotted arrows indicate that the same moment would be generated by an oppositely direct force acting on the other side of the axoneme. (**F)** The integrated moment along the length. (**G)** The pair of moments generate a bend. (**H)** Cartoon showing that bending moments come in pairs (unbalanced moments leads to rotations). (**I)** Tensile forces also come in pairs. Note 1. Assuming that the arm of DM8-dynein is parallel to the y -axis, then the moment it generates is positive: aj×−Fi=aFk , (where **i**, j and k are the unit vectors in the x , y and z directions). This moment, together with the moment −aFk generated by the DM3-dynein, creates a bend in the x - y plane. Note 2: Because the arms associated with the DM8-and DM3-dyneins are not (quite) opposite each other, the net moment generated when both are active (in the same section) do not (quite) cancel. However, the arm vectors $a_{n}=-a\sin\left(40n-20\right)j+a\cos\left(40n-20\right)k$ satisfy $\sum_{1}^{4}a_{n}+\sum_{6}^{9}a_{n}=ak$ . This is due to the absence of arms between DM5 and DM6. Thus, when all dyneins are active, the moment is ak×−fi=afj so there is no bending in the x - y plane. The bending in the y - z plane is blocked by the bridge, which prevents shear between doublets five and six.

Cilia and flagella are ancient organelles: all the major branches of eukaryotes include organisms with motile cilia and flagella (Jékely, 2009). The motile structure within the cilium is the axoneme, which has a diameter of about 200 nm (Afzelius, 1988) and can range in length from a few micrometers to over 1000 μm (Velho Rodrigues et al., 2021). Motile axonemes typically have a 9 + 2 architecture comprising nine circumferential doublet microtubules—each composed of a complete microtubule, the A-tubule, fused with an incomplete B-tubule—surrounding a central pair of single microtubules (Figures 1A,B). The microtubules form a scaffold that binds the axonemal dyneins, which drive motility, together with hundreds of other proteins that are essential for the assembly, structural integrity, and regulation of the axoneme (Pazour et al., 2005) (http://chlamyfp.org). Overall, the axoneme has a similar size to and number of proteins (encoded by different genes) as the mitochondrion (Calvo and Mootha, 2010), another evolutionarily ancient organelle. However, in contrast to mitochondria, where the essential features of the electrochemistry underlying aerobic respiration are understood (Berg, et al., 2019), the fundamental mechanochemistry underlying the motility of the axoneme is not well understood. This is because it is not known how mechanical forces generated by the dynein motors are coordinated into large-scale bending waves that propagate through the complicated axonemal structure. In this work, we address one part of this question: where in a beating cilium are the active dyneins located?

To understand how dyneins generate bending waves one must consider the geometry of the axoneme. The microtubule doublets are oriented such that the A-tubule is on the clockwise side (when looking from the base to the tip as in Figure 1B) and the B-tubule on the counterclockwise side: thus, the axoneme has approximate C9 symmetry with a rotation of 40° ( = 360 ÷ 9) from one doublet to the next. Axonemal dyneins are located along the axoneme with their tails anchored to the A-tubule of one doublet and their microtubule-binding domains (MBDs) interacting transiently with the B-tubule of the adjacent doublet (Figure 1A). The dyneins are ATPases, which bind adenosine triphosphate (ATP), hydrolyze it, and sequentially release the products inorganic phosphate and ADP (Ishibashi et al., 2020). This reaction is coupled to a sequence of conformational changes, which can be observed by electron microscopy (e.g., Burgess et al., 2003; Lin and Nicastro 2018). These conformational changes lead to dynein’s MBD attaching to the microtubule, the generation of force, which shears adjacent doublets, and the subsequent detachment from the microtubule. An important question in the field is: which conformational states of dynein correspond to the active, force-generating states, and which to inactive, non-force-generating states. This question is open: for example, a recent paper proposed that most of the dyneins in beating sea-urchin sperm are in force-generating states, and the bends are produced by inactivating dyneins a small fraction of dyneins at specific locations (Lin and Nicastro 2018). This view of the mechanism of bend formation and propagation differs from previously proposed mechanisms (Satir and Matsuoka, 1989), underscoring the importance of understanding the configurations of dyneins associated with force generation.

One way to answer this question is to deduce where, in a beating axoneme, the active dyneins are located. If one knew that dynein was active at a specific location (e.g., relative to the curvature of the axoneme, Figure 1A), then one could look at this location under the electron microscope and infer that the active state is the conformation of the dynein at this location. In this paper, we use a dynamical model of the axoneme, which we derive using a single-molecule approach, to predict the spatial relationship between motor activity and axonemal curvature. A key conclusion is that dynein activity and curvature do not colocalize. Furthermore, the spatial relationship between dynein force and axonemal bending depends on the ratio of the hydrodynamic and elastic forces, which in turn depends on the beat wavelength. Because they have shorter wavelengths than sperm, we argue that *Chlamydomonas* cilia are a better experimental model than sperm to identify dynein’s force-generating states by cryoem.

## Results

### General mechanism by which dyneins bend the axoneme

If the axonemal dyneins were not anchored to the A-tubule, they would walk along the B-tubule towards the base of the axoneme, where the microtubule’s minus end is located. Because the dyneins are anchored to the A-tubule, however, the force instead leads to sliding between adjacent doublets. This inter-doublet sliding in turn causes bending because there are mechanical constraints at the base of the axoneme that resist sliding there. The spatio-temporal coordination of the activity of the dyneins, through a mechanism that is not well understood (see e.g., Sartori et al., 2016b and references therein), gives rise to an approximately sinusoidal bending wave that travels along the axoneme and propels the axoneme through the fluid.

To connect motor activity with bending waves requires an equation of motion. Such an equation was first derived by Kenneth Machin (Machin, 1958), see also (Bayly and Wilson, 2014). It balances active forces against elastic forces (which oppose bending) and hydrodynamic forces (which oppose movement through the fluid). Using this equation, Machin deduced that active forces must be generated all along the flagellum; if motors were only active at the base, like the cracking of a whip, the amplitude would decay rapidly due to the damping from the fluid, and propagating bending waves would not be observed. In other words, “flagellum” (meaning whip in Latin) is a misnomer. Machin’s discovery is especially remarkable because dynein had not yet been discovered (Gibbons and Rowe, 1965) and he did not know that bending is driven by the sliding of the (nearly) incompressible microtubules (Satir, 1968).

Because Machin did not know how the forces were generated, he derived his equation using a continuum, non-molecular approach that is difficult to relate to our current understanding of motor proteins (e.g., (Ishibashi et al., 2020)). In this work, we rederive Machin’s equation by analyzing the forces generated by single dyneins. We show that the equation follows from just three molecular properties of dynein: 1) each active dynein generates a small bending moment, 2) a pair of active dyneins located on opposites sides of the axoneme and at different distances from the base bends the axoneme, 3) a difference in sliding force between two adjacent dyneins on one side of the axoneme produces a normal force that opposes hydrodynamic drag. Summing up these elementary interactions allows us to derive Machin’s equation and therefore deduce where active dyneins are located to produce the observed flagellar bends.

### How a pair of dyneins bends the axoneme

In this paper, we will assume that beating is driven by the outer-arm dyneins, which are anchored every 24 nm along the A-tubule and have two or three force-generating motor domains (depending on the species). This is a simplification as axonemes contain several different classes of inner-arm dyneins in addition to the outer-arm dyneins (Bui et al., 2008). However, there is functional redundancy among the dyneins: mutational studies show that *Chlamydomonas* cilia are still motile (though they beat more slowly) in the absence of the outer-arm dyneins or when individual classes of inner-arm dyneins are absent (Brokaw and Kamiya, 1987). Thus, given this redundancy, our simplification is likely to reasonable, at least at the level of analysis here.

Because the direction that dynein bends an axoneme depends on the doublet to which it is anchored, we need to use a numbering system for the doublets. The doublets (and associated dyneins) are numbered in Figure 1B according to the convention for sperm (Afzelius, 1988). Doublet microtubule 1 (DM1) is defined as the doublet that lies on the line that bisects the central pair. On the opposite side to DM1 there is usually a bridge that connects doublets five and six, between which the outer-arm dyneins are missing. In the unicellular alga *Chlamydomonas reinhardtii*, the numbering differs (Hoops and Witman, 1983): the bridge is between *Chlamydomonas* doublets one and 2 (cDM1 and cDM2), and cDM5 is equivalent to DM1 in sperm. The absence of dyneins between DM5 and DM6 in sperm (and cDM1 and cDM2 in *Chlamydomonas*) and the presence of the bridge, which presumably impedes sliding, tends to keep the axonemal beat in the x−y plane (see Note 2 in the legend to Figure 1).

To analyze mathematically how the dyneins bend the axoneme, we need to define a coordinate system. In the right-handed coordinate system shown at the bottom of Figures 1A,B, the x -axis is parallel to the axis of the axoneme and the y -axis points into the reverse bend (defined as the bend which has the bridge on the inside Lin et al., 2014); a useful mnemonic is RBI—the Reverse bend has the Bridge on the Inside. An axoneme shape corresponds to a curve y(x) . The z -axis points out of the page in Figure 1A and upwards in Figure 1B. This coordinate system defines the sign convention for curvature ( $≅d^{2}y/dx^{2}$ ) and bending moment (see below). The origin of the coordinate system is at the base of the axoneme ( x=0 , y=0 ) and the tip of a straight axoneme is at ( x=L , y=0) , where L is the length.

To simplify the analysis, we focus only on the dyneins between doublets three and four (DM3 filled black) and those between eight and nine (DM8 filled gray) (Figure 1C); these are the main drivers for bending in the x - y plane, with the other dyneins generating moments that make smaller contributions to the bend (see Notes 1 and 2 in the legend to Figure 1). The arms extend approximately parallel to the y -axis and are spaced with a period δ= 24 nm along the length of the doublet as shown in Figure 1D, which is in the same orientation as Figure 1A. The dyneins walk towards the minus ends of the microtubules, which are located towards the cell body ( x=0 ). Therefore, they generate minus-end-directed forces, indicated by the downward arrows. Following this sign convention, the dynein force is −F , where F is positive. Single outer-arm dyneins can generate forces up to 5 pN (Hirakawa et al., 2000). Because the inter-dynein spacing (24 nm) is very small compared to the length of the bends (typical wavelengths are λ∼10 μm), we can define a force density per unit length, −f , where f=F/δ .

The next step is to calculate the bending moments generated by the dyneins. In a beating axoneme, dynein forces vary with position (both along the length and on different sides of the axoneme) and time. It is instructive, however, to start with a simple scenario in which only two dyneins are active and the activity does not change in time. The dyneins are shown in Figure 1E: one at the lower left (gray MTB) and the other at the upper right (black MTB). The lower dynein is anchored to the A-tubule of DM8 and interacts with the B-tubule of DM9 and generates a downward force with a moment arm extending to the left (which is positive in our coordinate system). The magnitude of the moment arm, a≅ 30 nm, corresponds approximately to the distance between the doublets, though the precise length depends on the molecular structure of dynein and how it generates force. The DM8 dynein generates a small counterclockwise (positive) moment $M_{l}=aF_{l}$ , where $-F_{l}$ is the dynein force on the left (see Note 1 in Figure 1 legend for the definition of the sign of the moment). Note that if the dynein immediately to the right of this dynein (on the opposite side of the midline) were also active and generating a downward force, then the moments would cancel and there would be no net moment (see Note 2 in Figure 1 legend for a more precise statement). This illustrates that a net bending moment requires an imbalance of forces across the axoneme. The dynein anchored to DM3 and interacting with DM4 with its black MTB (Figure 1E, upper right) also generates a downward force, but the moment arm extends to the right (i.e., the negative direction); this dynein generates a small clockwise (negative) moment $M_{r}=-aF_{r}$ . Therefore, in between this pair of dyneins is a region where the moment, $M=aF=a\left(F_{l}-F_{r}\right)$ , is positive (Figure 1F). This moment bends the intervening axoneme (Figure 1G).

### The location and size of the bends generated by the dyneins

The magnitude of the bend produced by the pair of moments can be calculated using the “beam” equation, M=−κC , where κ is the flexural rigidity of the axoneme and $C=d^{2}y/dx^{2}$ is the curvature (Howard 2001). This equation is a consequence of Euler–Bernoulli beam theory and serves as a definition of the flexural rigidity. In accordance with our sign convention, the curvature of the bend in Figure 1G is negative (the angle decreases as x -increases).

The beam equation is analogous to Hooke’s equation for the extension of a spring: F=−kx , where k is the stiffness and x is the extension. To create a bend, two equal and opposite moments are needed (Figure 1H); a single bending moment will cause an object to spin. This is analogous to stretching a spring: two equal and opposite forces are needed (Figure 1I): a single force will cause an object to translate.

If only one dynein is actively generating force, then to get a bend, the moment must be balanced at the base (or tip) of the axoneme, for example by restricting sliding at the basal body or transition zone. If the moment is not balanced at the base (or along the length), then the doublets will slide apart without bending, as observed when the basal restriction to sliding is digested away with proteases (Summers and Gibbons, 1971). In addition to a sliding constraint at the base, bending as shown in Figure 1G requires an additional constraint: DM8 and DM3 must bend together otherwise the DM8-DM9 pair would bend in one direction and DM3-DM4 pair would bend in the other. This constraint is supplied by the radial spokes and additional electrostatic interactions between the doublets that keep the spacing between doublets fixed and maintain the circular cross-section of the axoneme.

A key finding of this analysis is that the location of the bend differs from the location of the active dynein motors that cause it. Figures 1E–G show this: the bend occurs between the active dyneins, and the curvature outside the dyneins is zero.

How big is the bend? Given that the axoneme contains 20 microtubules (9 doublets plus two making up the central pair), we expect that flexural rigidity to be ≥20 times that of a single microtubule (it could be much larger if there is resistance to inter-doublet sliding, which occurs in the absence of ATP and the motors are in rigor, (Howard, 2001)). Therefore, we expect the flexural rigidity to be at least 500 × 10^–24^ N∙m^2^ or 500 pN μm^2^ (using the flexural rigidity of a single microtubule in (Howard, 2001)). This flexural rigidity agrees with experimental measurements on intact axonemes (e.g., 800 pN μm^2^ in (Xu et al., 2016)). According to the beam equation, therefore, a single pair of dyneins is expected to generate only a very slight bend with curvature 0.0002 μm^−1^, corresponding to a radius of curvature of about 5,000 μm ( $\left|C\right|=aF/\kappa =0.03\;\mu m\times 5\;pN÷800\;pN∙{\mu m}^{2}$ ). Thus, the large bends observed in beating axonemes (radius of curvature on the order of 1–10 μm) must be generated by hundreds of dyneins within each wavelength.

### Balancing motor forces and bending forces in a static axoneme

To find a general relationship between the distribution of motor forces and the curvature of an axoneme, we derive the static version of Machin’s equation. We start with the beam equation: M(x)=−κC(x) . To calculate the total bending moment, M , at position x , we need to add up all the moment densities along the length: $M\left(x\right)=\int_{0}^{x}m\left(x^{\prime }\right)dx^{\prime }=\int_{0}^{x}a∙f\left(x^{\prime }\right)dx^{\prime }$ , where $f=f_{l}-f_{r}$ is the differential force density across the axoneme ( $f_{l}=F_{l}/\delta ,f_{r}=F_{r}/\delta )$ . If we assume that the amplitude of the beat is small, then the curvature is approximately $C\left(x\right)≅d^{2}y/dx^{2}$ . We therefore obtain *M* $\left(x\right)=\int_{0}^{x}a∙f\left(x^{\prime }\right)dx^{\prime }=-\kappa C=-\kappa d^{2}y/dx^{2}$ . Differentiation gives

$$af\left(x\right)=-\kappa \frac{d^{3}y}{dx^{3}}\left(x\right)$$ (1)

**FIGURE 2:**
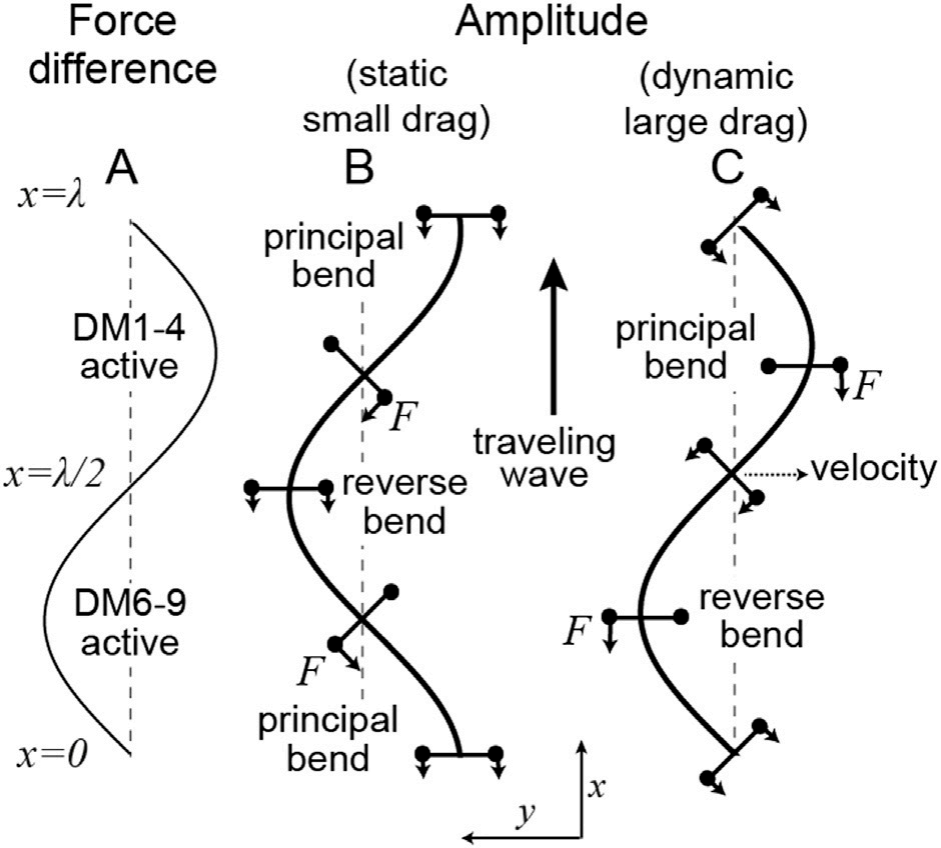
Location of motor forces relative to the axonemal shape in the static (small drag) and dynamic (large drag) limits (**A)** The difference in forces between those generated by dyneins anchored on DM6-9 (defined as positive) and those anchored on DM1-4. (**B)** In the static case ( Ma=0 , see Eq. 6 for the definition of the Machin number Ma ), the amplitude lags the force. The negative bend is associated with the activity of DM6-9 dyneins in front of the bend and the DM1-4 dyneins behind the bend. (**C)** In the hydrodynamic limit ( Ma=∞ ), the amplitude is in phase with the force. In this case, proximal DM6-9 dyneins and distal DM1-4 dyneins will produce a negative (reverse) bend in the intervening straight region. The bending wave travels upwards as indicated by the arrow. The forces and moment arms are indicated in B and C.

This equation relates the differential dynein force density to the change in curvature in a static (unmoving) axoneme. If the forces are distributed along the axoneme as f(x)∝sin(2πx⁄λ) as shown in Figure 2A (i.e., $f_{r}=f_{l}$ at x=0 and DM6-9 maximally active at x=λ /4), then the amplitude can be obtained by triple integration of Eq. (1): y(x)∝−cos(2πx⁄λ) . The curvature, $C=d^{2}y/dx^{2}=\cos\left(2\pi x/\lambda \right)$ , is therefore λ /4 (ninety degrees) out of phase with the motor activity and the principal bend (maximum positive curvature) occurs before the force peaks. The motor forces have maximum amplitude at each end of the bend. This is analogous to the case illustrated in Figures 1E–G. Furthermore, where the curvature is maximum (and minimum) there is no differential motor activity! Thus, motor activity and bending are not co-localized. This is a key finding.

### Balancing motor forces and hydrodynamic forces

**FIGURE 3:**
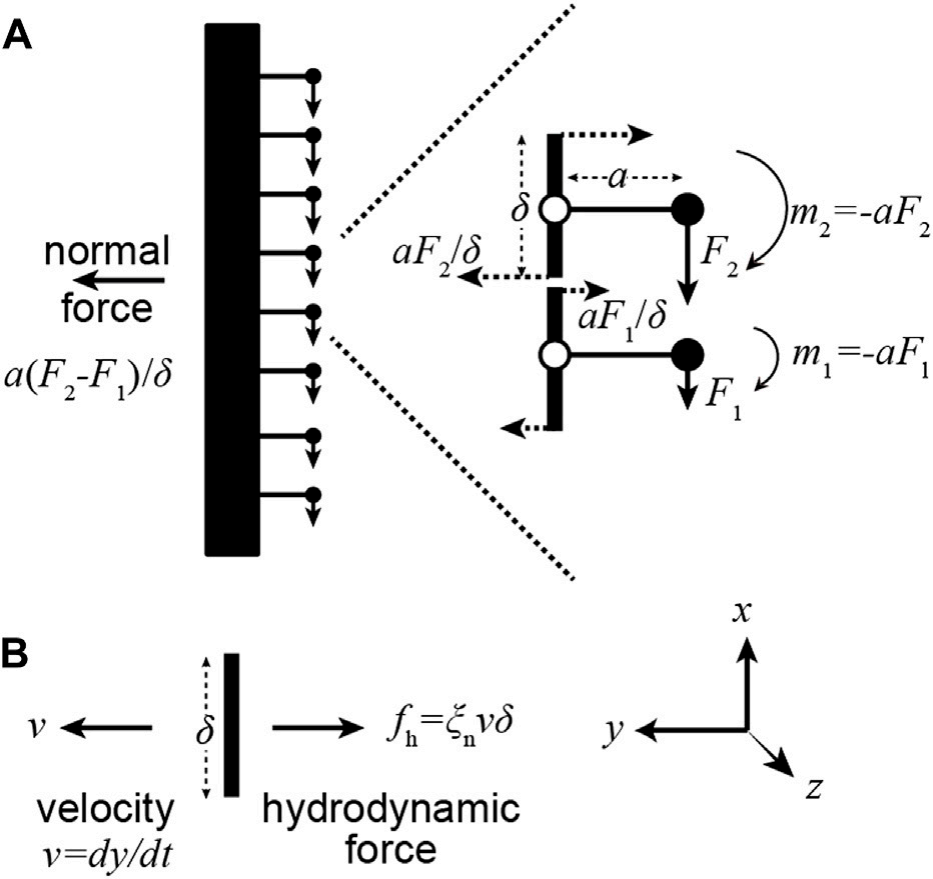
A gradient of motor forces produces a normal force. (**A)** Consider a pair of adjacent dyneins in which the upper dynein generates a larger force ( $F_{2}>F_{1}$ ). The upper moment produces a larger leftward force than the lower moment produces a rightward force. The net force is proportional to the gradient. (**B)** The net rightward normal force can balance the leftward hydrodynamic force when the axonemal segment moves to the left.

As an axoneme swims, the movement of each increment of length along the flagellum is opposed by viscous forces from the surrounding fluid. In a moving flagellum, therefore, the motor forces must also balance hydrodynamic forces, which are normal to the axis of the axoneme. That the motor moments produce normal forces can be seen with the help of Figure 3. At the ends of each increment of length (length δ in this example), the bending moment generated by the dynein also generates a normal force aF/δ . If each dynein generates the same force, and therefore the same bending moment, then the net normal force is zero. However, if there is a gradient of dynein forces, then the net normal force is non-zero: it is a∆F/δ , where $∆F=F_{2}-F_{1}$ . This force can balance the hydrodynamic force acting on the segment: $-\xi_{n}v_{n}\delta$ , where $v_{n}≅dy/dt$ is the normal velocity of the increment and $\xi_{n}$ is the normal drag coefficient per unit length (Friedrich et al., 2010). Noting that F/δ is the force density, f , and that the change in f , namely ∆f , occurs over distance ∆x=δ , we obtain:

$$a\frac{\partial f}{\partial x}\left(x\right)=-\xi_{n}\frac{\partial y}{\partial t}\left(x\right)$$ (2)

In other words, gradients of motor forces generate normal forces. This equation holds in the limit that the elastic forces are small, in which case the motors only balance hydrodynamic forces. This equation can also be derived by balancing the motor force against all the moments generated by the hydrodynamic forces at locations ≥x and differentiating (Howard 2001; Appendix 6.2); we have derived it this way to more directly indicate that pairs of dyneins generate the normal force.

In the limit where the hydrodynamic forces dominate, Eq. 2 specifies how a force density, which varies in space and time, determines the shape of the axoneme. For example, let the force be a traveling wave, f(x,t)∝sin[(2π(x⁄λ−νt)] where λ is the wavelength and ν is the frequency (in Hz). f(x,0) is plotted in Figure 2A. The speed is λν (traveling from base to tip). In this case, the amplitude y(x,t)∝sin[(2π(x⁄λ−νt)] is in phase with the force (Figure 2C). The curvature ( C=−sin[(2π(x⁄λ−νt)] is therefore 180° out of phase with the motor activity. The sign of the motor activity can be understood by realizing that when hydrodynamic damping dominates, the place with highest velocity needs to be balanced by the motor moments. This place is the straight region where x=λ/2 (which will become the reverse bend). Thus, motors anchored to DM6-9 need to be active before the straight part ( x=λ/4 ) and the motors anchored to DM1-4 need to be active after the straight part ( x=3λ/4 ).

That the dyneins anchored to DM6-9 are active in the reverse bend is counter-intuitive. This is because, if only dynein DM8 were active and there was no basal sliding (i.e., the negative moment is at the base and not at DM3 in Figure 1G), then a principal bend, not a reverse bend, would be generated.

### Balancing motor forces with elastic and hydrodynamic forces in a moving axoneme

In the prior sections we have considered the limiting cases in which motor forces balance only elastic forces (negligible hydrodynamic forces) or they balance only hydrodynamic forces (negligible elastic forces). In general, the motors balance the sum of the hydrodynamic forces and the elastic forces:

$$a\frac{\partial f}{\partial x}=-\xi_{n}\frac{\partial y}{\partial t}-\kappa \frac{\partial^{4}y}{\partial x^{4}}$$ (3)

This is Machin’s equation (Eq. 16) in Machin 1958; Machin’s parameter B is the integrated bending moment, M=a∫fdx , in our notation). This equation is equivalent to Eq. 16 in Camalet and Jülicher (2000) and Equation (14) Sartori et al. (2016b) if f is replaced by −f (due to the different sign convention used here). If Eq. 3 is differentiated again with respect to x , and we substitute ψ for dy/dx ( ψ is tangent angle), and equate x with arc length s , then we obtain a somewhat more general equation (it holds for small angles ψ and not just small amplitudes y ) used in (Riedel-Kruse et al., 2007). Eq. 3 can also be written

$$m_{motors}+m_{drag}+m_{elastic}=0$$ (4)

showing that the moment densities are balanced.

In summary, we have shown that the Machin’s equation can be derived by summing up the forces generated by pairs of dynein molecules.

### Predicted locations of active dyneins in beating axonemes

Machin’s equation can be used to predict where the force is being generated in a beating axoneme. If the amplitude y(x,t) is known in space and time, then we can use Eq. 3 to deduce the force f(x,t) . Note that if we have a “motor model”, meaning that we know how the activity of the motors depends on the shape (i.e., $f\left(y,\partial y/\partial x,\;\partial^{2}y/\partial x^{2}\ldots \right)$ ) then Machin’s equation becomes a dynamical system that can integrated (with appropriate boundary conditions) to predict self-organized waveforms. This approach, which entails completing a feedback loop in which motor activity bends the axoneme and the bending of the axoneme feeds back on motor activity, has been used in several earlier studies (see (Sartori et al., 2016b) for references). In this work, we ask the simpler question: given a shape, what is the force profile?

The force profile is readily deduced in the special case where the shape of a beating cilium or flagellum resembles a sinusoid, as is often the case. This so-called travelling wave, with amplitude y(x,t)∝sin[(2π(x⁄λ−νt)] , is an approximation that holds in the limit that the axoneme is infinitely long, in which case the boundary conditions can be neglected. The wave travels form base to tip with velocity λν . Traveling waves afford a particularly simple relationship between the shape and the motor force: the motor activity is also sinusoidal (seen by substitution into Eq. (3)) with a simple phase shift:

f(x,t)=sin⁡⁡[2π(x⁄λ−νt)+ϕ] (5)

where the phase is

ϕ=arctan(1/Ma); (6)

$$Ma=\frac{\nu \xi_{n}\;\lambda^{4}}{{\left(2\pi \right)}^{3}\;\kappa }$$ (7)

is the Machin number, which quantifies the ratio of the viscous forces to the elastic forces for a traveling wave (Geyer et al., 2022). Eq. (6) shows that the Machin number can also be defined from the phase between the force and the amplitude for a traveling wave. ϕ>0 means that the force leads the amplitude in space and lags the curvature in space. Equivalently, it means that the force leads the curvature in time: the place where the force is high is where the curvature will become high, as noted in Sartori et al. (2016b).

**FIGURE 4:**
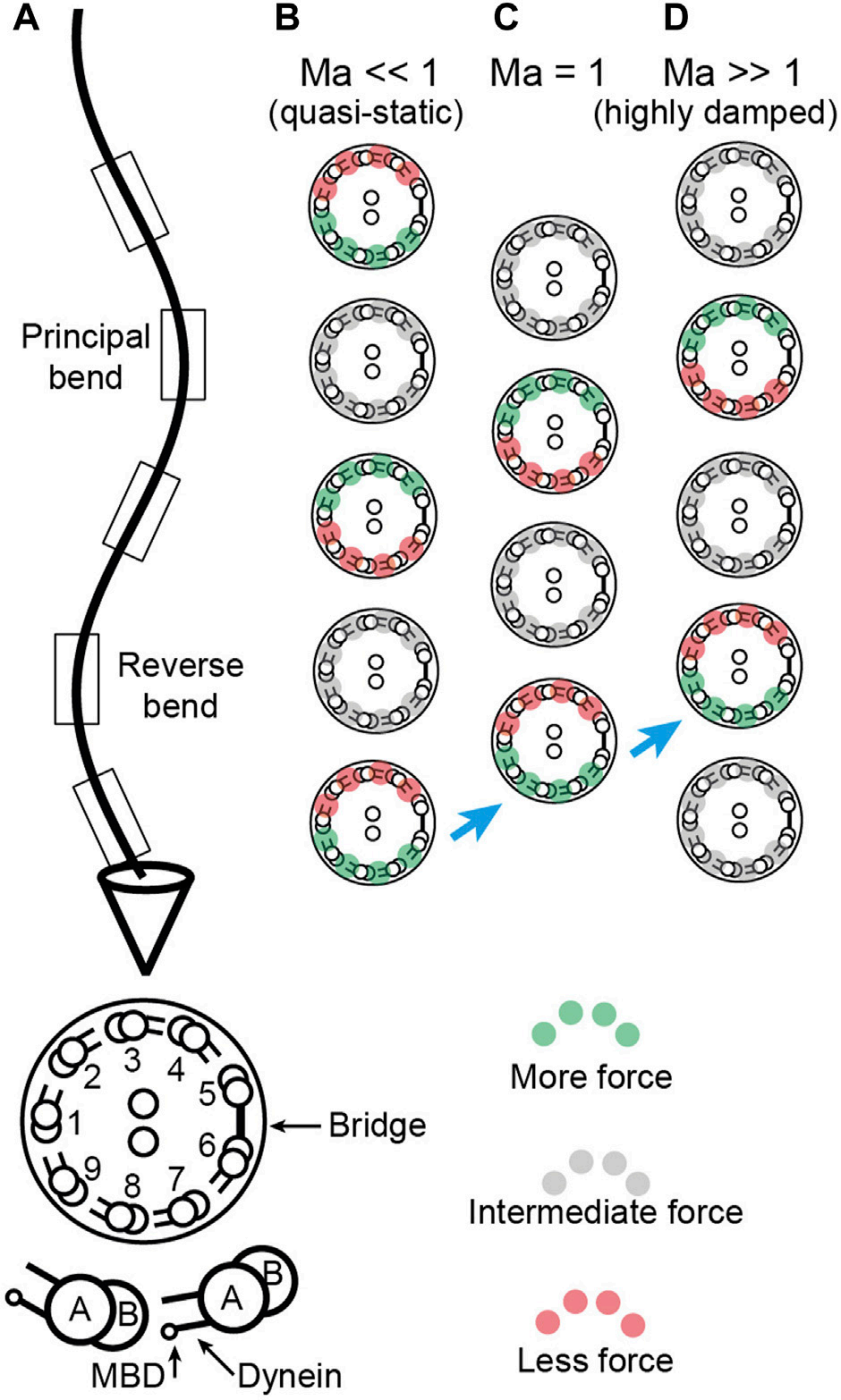
Location of sliding forces in a beating axoneme. **(A)** View of a beating sperm. The section is viewed from the base. The reverse bend is defined as having the bridge on the inside. This view can be transformed to that of the *Chlamydomonas* axoneme in Sartori et al. (2016b) Figure 1 by rotating the axoneme 180° about its long axis and using the *Chlamydomonas* numbering, again with the bridge on the inside of the reverse bend. (**B-D)** Location of active, force-generating dyneins for Machin numbers ≪1 (B, small hydrodynamic and large elastic forces), =1 (C, equal hydrodynamic and elastic forces) and ≫1 (D, large hydrodynamic and small elastic forces). The green dyneins exert high force, the gray dyneins exert intermediate force and the red exert low force. The blue arrows indicate the increasing spatial lag of the dyneins relative to the curvature as the Machin number increases.

When Ma≪1 (which occurs when the wavelength is small, the frequency low, the drag is small, the flexural rigidity is large), the phase is approximately π/2 . In this case, the curvature leads the motor force in space by ∼π/2 (Figure 2B). This is illustrated in (Figure 4B). Equivalently, the curvature lags the motor force in time by ∼π/2 : this means that the place where the motors are maximally active is where the principal curvature is increasing and will become the principal bend. When Ma≫1 (which occurs when the wavelength is long, the frequency high, the drag is large, the flexural rigidity is small), the phase is shifted towards zero (Figure 4D): now the amplitude is in phase with the force and the curvature lags the motor force (in time) by a phase that approaches π . At an intermediate Machin number, Ma=1 , the curvature lags the force by, 3π/4 intermediate between π/2 and π (Figure 4C). The additional phase lag in the presence of drag makes sense because increased damping generally causes an increase in the temporal lag between a response and the force that produces it (for example, a damped spring). Figures 4B,C shows this increasing temporal lag of the curvature behind the force as an increasing spatial lag of the force behind the curvature: the maximum force is further and further behind the curvature (i.e., towards the tip) as Ma increases (blue arrows).

## Discussion

We have derived Machin’s equation by considering the moments generated by pairs of dyneins. When a pair of dyneins exert equal and opposite moments (forces in the same direction but on opposite sides of the axoneme), then the intervening axoneme will bend if the dyneins are at different axial positions along the axonemal length (Figure 1). There is no bend if there are at the same axial position, showing that bending requires differential activity of dyneins across the axoneme. When a pair of dyneins on the same side of the axonemal section, but at different positions along the axoneme, exert different forces, they generate a force orthogonal to the axis of the axoneme. This force can oppose hydrodynamic forces (Figure 3). Integrating the forces generated by such pairs of dyneins leads to Machin’s equation (Eq. 3). This equation, in turn, predicts that a traveling wave of dynein force-generating activity will generate a traveling wave of curvature (Figure 2).

Two key findings follow from our analysis. The first is that the force and curvature are not colocalized (i.e., they are not in phase). And the second is that the phase shift between the force and the curvature depends on the relative amplitude of the hydrodynamic forces and the elastic forces. The ratio of these forces is the Machin number, Ma (Eq. 7). If we know the Machin number, Machin’s equation allows us to predict where, relative to the curvature, the dyneins must be active.

The clearest predictions are for short-wavelength cilia such as those of the unicellular alga *Chlamydomonas*. *Chlamydomonas* has a beat wavelength of ∼10 μm, similar to the length of the axoneme. Several lines of evidence suggest that Ma≪1 for *Chlamydomonas* (Geyer et al., 2022). First, the values of the parameters that make up Machin’s number suggest that Ma is between 0.02 and 0.14. Second, the waveforms are well-described by a dynamic model that has small Ma . And third, the ATPase rate of axonemes increases in proportion to beat frequency, as predicted if elastic dissipation is larger than hydrodynamic dissipation (Chen et al., 2015). Ma being very small has the advantage that the phase is close to π/2 , and does not depend much on the exact value of Ma . For example, tan^−1^ (1/0.02) ≅ 89° while tan^−1^ (1/0.14) ≅ 82°; a seven-fold change in Ma leads to a phase shift of only 7°. Therefore, the small Ma makes *Chlamydomonas* suitable for these measurements. Furthermore, *Chlamdomonas* has an almost planar beat, which also makes it suitable (the small deviations from planarity lead to helical beats but only over long distances, Sartori et al., 2016a). A potential disadvantage of *Chlamydomonas* is that the wild-type cells have asymmetric beats, which are unsuitable for these measurements as the combined static and dynamic curvatures are likely to confound the analysis. The *mbo*2 mutant, however, has a symmetric beat, which is similar to the dynamic component of the wild-type beat (Geyer et al., 2016) and *mbo2* cilia have similar lengths and wavelengths to wild-type cilia. Thus, the *mbo*2 mutant of *Chlamydomonas* is a good preparation for cryoEM studies to identify which conformations of axonemal dyneins are the force-generating ones.

Sperm flagella are less suitable for these measurements than *Chlamydomonas* cilia. This is because the Machin number is on the order of unity for sperm from sea-urchin and mouse, using parameters from Velho (Rodrigues et al., 2021). The phase associated with this value of the Machin number is highly dependent on the exact value of Ma , which depends on parameters whose values are uncertain. Furthermore, other factors such as increased hydrodynamic friction during the preparation of samples for cryoEM (e.g., close proximity to the grid or air-water interface, and increased viscosity due to freezing) may lead to additional changes in the Machin number that make prediction of the phase uncertain. For these reasons, the recent cryoEM measurements of the dynein conformations in sea-urchin sperm (Lin and Nicastro 2018) are expected to be difficult to correlate with curvature. A preliminary analysis with Ma=1 , predicts a phase shift of the curvature relative to the force equal to 135°, which is quite different to the 0° expected from Lin and Nicastro’s assumption that the pre-power-stroke states are the force generating ones. In other words, this assumption is not consistent with the analysis presented here. It is interesting to note that Lin and Nicastro’s assumption is that the force-generating dyneins in the positive bend are DM6-9; however, as we pointed out in the section “Balancing motor force against hydrodynamic forces”, when hydrodynamic forces dominate, the DM6-9 dyneins are active in the negative bend, which is counter-intuitive for the reason we outlined in the earlier section. Further theoretical and experimental work is needed to resolve this discrepancy.

## Data Availability

The original contributions presented in the study are included in the article. Further inquiries can be directed to the corresponding author.

## References

[B1] Afzelius B. A. (1988). On the numbering of peripheral doublets in cilia and flagella. Tissue Cell 20, 473–475. 10.1016/0040-8166(88)90078-X 18620237

[B2] Bayly P. v. Wilson K. S. (2014). Equations of interdoublet separation during flagella motion reveal mechanisms of wave propagation and instability. Biophys. J. 107, 1756–1772. 10.1016/J.BPJ.2014.07.064 25296329 PMC4190657

[B3] Berg J. M. Tymoczko J. L. Gatto G. J. Jr. Stryer L. (2019). Biochemistry. Dallas, Texas: Freeman.

[B4] Brokaw C. J. Kamiya R. (1987). Bending patterns of Chlamydomonas flagella: IV. Mutants with defects in inner and outer dynein arms indicate differences in dynein arm function. Cell Motil. Cytoskelet. 8, 68–75. 10.1002/cm.970080110 2958145

[B5] Bui K. H. Sakakibara H. Movassagh T. Oiwa K. Ishikawa T. (2008). Molecular architecture of inner dynein arms *in situ* in Chlamydomonas reinhardtii flagella. J. Cell Biol. 183, 923–932. 10.1083/jcb.200808050 19029338 PMC2592835

[B6] Burgess S. A. Walker M. L. Sakakibara H. Knight P. J. Oiwa K. (2003). Dynein structure and power stroke. Nature 421, 715–718. 10.1038/nature01377 12610617

[B7] Calvo S. E. Mootha V. K. (2010). The mitochondrial proteome and human disease. Annu. Rev. Genomics Hum. Genet. 11, 25–44. 10.1146/ANNUREV-GENOM-082509-141720 20690818 PMC4397899

[B8] Camalet S. Jülicher F. (2000). Generic aspects of axonemal beating. New J. Phys. 2, 324. 10.1088/1367-2630/2/1/324

[B9] Chen D. T. N. Heymann M. Fraden S. Nicastro D. Zvonimir Dogic Z. (2015). ATP consumption of eukaryotic flagella measured at a single-cell level. Biophys. J. 109, 2562–2573. 10.1016/j.bpj.2015.11.003 26682814 PMC4699893

[B10] Friedrich B. M. Riedel-Kruse I. H. Howard J. Jülicher F. (2010). High-precision tracking of sperm swimming fine structure provides strong test of resistive force theory. J. Exp. Biol. 213, 1226–1234. 10.1242/jeb.039800 20348333

[B11] Geyer V. F. Howard J. Sartori P. (2022). Ciliary beating patterns map onto a low-dimensional behavioural space. Nat. Phys. 18, 332–337. 10.1038/s41567-021-01446-2 PMC12242449 40642382

[B12] Gibbons I. R. Rowe A. J. (1965). Dynein: A protein with adenosine triphosphatase activity from cilia. Science 149, 424–426. 10.1126/science.149.3682.424 17809406

[B13] Hirakawa E. Higuchi H. Toyoshima Y. Y. (2000). Processive movement of single 22S dynein molecules occurs only at low ATP concentrations. Proc. Natl. Acad. Sci. U. S. A. 97, 2533–2537. 10.1073/pnas.050585297 10706634 PMC15963

[B14] Hoops H. J. Witman G. B. (1983). Outer doublet heterogeneity reveals structural polarity related to beat direction in Chlamydomonas flagella. J. Cell Biol. 97, 902–908. 10.1083/jcb.97.3.902 6224802 PMC2112583

[B15] Howard J. (2001). Mechanics of motor proteins and the cytoskeleton. Sunderland, MA: Physics Today.

[B16] Ishibashi K. Sakakibara H. Oiwa K. (2020). Force-generating mechanism of axonemal dynein in solo and ensemble. Int. J. Mol. Sci. 21, E2843. 10.3390/ijms21082843 32325779 PMC7215579

[B17] Jékely G. (2009). Evolution of phototaxis. Philos. Trans. R. Soc. Lond. B Biol. Sci. 364, 2795–2808. 10.1098/RSTB.2009.0072 19720645 PMC2781859

[B18] Lin J. Nicastro D. (2018). Asymmetric distribution and spatial switching of dynein activity generates ciliary motility. Science 360, eaar1968. 10.1126/science.aar1968 29700238 PMC6640125

[B19] Lin J. Okada K. Raytchev M. Smith M. C. Nicastro D. M. (2014). Structural mechanism of the dynein power stroke. Nat. Cell Biol. 16, 479–485. 10.1038/ncb2939 24727830 PMC4102432

[B20] Machin K. (1958). Wave propagation along flagella. J. Exp. Biol. 35, 796–806. 10.1242/jeb.35.4.796

[B21] Pazour G. J. Agrin N. Leszyk J. Witman G. B. (2005). Proteomic analysis of a eukaryotic cilium. J. Cell Biol. 170, 103–113. 10.1083/JCB.200504008 15998802 PMC2171396

[B22] Riedel-Kruse I. H. Hilfinger A. Howard J. Jülicher F. (2007). How molecular motors shape the flagellar beat. HFSP J. 1, 192–208. 10.2976/1.2773861 19404446 PMC2640991

[B29] Sartori P. Geyer V. F. Howard J. Jülicher J. (2016a). Curvature regulation of the ciliary beat through axonemal twist. Phys. Rev. E. 94, 042426. 27841522 10.1103/PhysRevE.94.042426

[B23] Sartori P. Geyer V. F. Scholich A. Jülicher F. Howard J. (2016b). Dynamic curvature regulation accounts for the symmetric and asymmetric beats of Chlamydomonas flagella. Elife 5, e13258. 10.7554/eLife.13258 27166516 PMC4924999

[B24] Satir P. Matsuoka T. (1989). Splitting the ciliary axoneme: Implications for a “switch-point’’ model of dynein arm activity in ciliary motion. Cell Motil. Cytoskelet. 14, 345–358. 10.1002/cm.970140305 2531043

[B25] Satir P. (1968). Studies on cilia. 3. Further studies on the cilium tip and a "sliding filament" model of ciliary motility.. J. Cell Biol. 39, 77–94. 10.1083/jcb.39.1.77 5678451 PMC2107504

[B26] Summers K. E. Gibbons I. R. (1971). Adenosine triphosphate-induced sliding of tubules in trypsin-treated flagella of sea-urchin sperm.. Proc. Natl. Acad. Sci. U. S. A. 68, 3092–3096. 10.1073/pnas.68.12.3092 5289252 PMC389597

[B27] Velho Rodrigues M. F. Lisicki M. Lauga E. (2021). The bank of swimming organisms at the micron scale (BOSO-Micro). PLOS ONE 16, e0252291. 10.1371/JOURNAL.PONE.0252291 34111118 PMC8191957

[B28] Xu G. Wilson K. S. Okamoto R. J. Shao J. Y. Dutcher S. K. Bayly P. V. (2016). Flexural rigidity and shear stiffness of flagella estimated from induced bends and counterbends. Biophys. J. 110, 2759–2768. 10.1016/j.bpj.2016.05.017 27332134 PMC4919507

